# Hypnosis for pain and anxiety management in cognitively impaired older adults undergoing scheduled lumbar punctures: a randomized controlled pilot study

**DOI:** 10.1186/s13195-022-01065-w

**Published:** 2022-09-02

**Authors:** Pauline Courtois-Amiot, Anaïs Cloppet-Fontaine, Aurore Poissonnet, Elodie Benit, Muriel Dauzet, Agathe Raynaud-Simon, Claire Paquet, Matthieu Lilamand

**Affiliations:** 1 AP-HP. Nord, Geriatric Department, Bichat Hospital, 46 rue Henri Huchard, 75018 Paris, Cedex 18 France; 2 Gérond’If, Gérontopôle d’Ile-de-France, 33 rue du Fer à Moulin, 75005 Paris, France; 3 AP-HP. Nord, Geriatric Day Hospital, Bretonneau Hospital, 23 rue Joseph de Maistre, 75018 Paris, France; 4 Université de Paris, Paris, France; 5 INSERM 1144 Research Unit, Paris, France; 6 AP-HP. Nord, Cognitive Neurology Center, Lariboisière Fernand-Widal Hospital, 200 rue du Faubourg Saint Denis, 75010 Paris, France

**Keywords:** Hypnosis, Lumbar puncture, Older adults, Pain, Anxiety

## Abstract

**Background:**

Core cerebrospinal fluid (CSF) amyloid and tau biomarker assessment has been recommended to refine the diagnostic accuracy of Alzheimer’s disease. Lumbar punctures (LP) are invasive procedures that might induce anxiety and pain. The use of non-pharmacological techniques must be considered to reduce the patient’s discomfort, in this setting. The objective of this study was to examine the efficacy of hypnosis on anxiety and pain associated with LP.

**Methods:**

A monocentric interventional randomized-controlled pilot study is conducted in a university geriatric day hospital. Cognitively impaired patients aged over 70 were referred for scheduled LP for the diagnostic purpose (CSF biomarkers). The participants were randomly assigned either to a hypnosis intervention group or usual care. Pain and anxiety were both self-assessed by the patient and hetero-evaluated by the operator.

**Results:**

We included 50 cognitively impaired elderly outpatients (women 54%, mean age 77.2 ± 5.0, mean Mini-Mental State Examination score 23.2 ± 3.5). Hypnosis was significantly associated with reduced self-assessed (*p* < 0.05) and hetero-assessed anxiety (*p* < 0.01). Hetero-evaluated pain was significantly lower in the hypnosis group (*p* < 0.05). The overall perception of hypnosis was safe, well-accepted, and feasible in all the participants of the intervention group with 68% perceiving the procedure as *better* or *much better* than expected.

**Conclusions:**

This pilot study suggested that hypnosis was feasible and may be used to reduce the symptoms of discomfort due to invasive procedures in older cognitively impaired patients. Our results also confirmed the overall good acceptance of LP in this population, despite the usual negative perception.

**Trial registration:**

ClinicalTrials.gov NCT04368572. Registered on April 30, 2020.

## Background

Alzheimer’s disease (AD) is the most prevalent cause of major neurocognitive disorders worldwide and mostly affects elderly subjects [1]. Core cerebrospinal fluid (CSF) amyloid and tau biomarker assessment has been recommended to refine the diagnostic accuracy of AD [2, 3]. As the population ages, and the incidence of cognitive disorders is on the rise, this diagnostic procedure will be increasingly used. Lumbar puncture (LP) is a common minimally invasive procedure and may be performed by physicians from various specialties. Complications are rare, mostly minor (postdural puncture headache), and significantly reduced by the use of atraumatic needles [3, 4]. Nevertheless, the general picture of LP and associated side effects remains negative, although generally safe and well-tolerated [3, 5–7]. The use of non-pharmacological techniques, before or during the procedure, must be considered to reduce LP-related anxiety and pain, so that its acceptance in routine clinical practice would be improved.

The American Psychological Association defines hypnosis as “a state of modified consciousness involving focused attention and reduced peripheral awareness characterized by an enhanced capacity for response to suggestion” [8]. Hypnosis associates a set of techniques that can be used independently of each other, making this tool a multifaceted therapy: e.g., hypnoanalgesia for the management of pain, hypnosedation used mainly in anesthesia, and hypnotherapy for psychotherapeutic applications. In a 2015 report from the French National Institute of Health and Medical Research (INSERM), the effectiveness of hypnosis has been demonstrated for reducing the consumption of analgesics or sedatives during invasive procedures, in surgery or in interventional radiology [9].

The absence of cognitive impairment and good concentration skills have been considered as prerequisites for an effective practice of hypnosis. Nevertheless, some scientific publications support the possible use of hypnosis in the elderly. In fact, age would not modify hypnotisability or the effectiveness of hypnosis to manage anxiety and pain during an interventional radiology procedure [10]. In a small-sampled pilot study of cognitively impaired individuals, hypnosis was associated with an improvement in the quality of life, in subjects with dementia, without any specific limitation due to cognitive disorders [11]. Thus, numerous benefits may be highlighted in this population: non-invasive, acceptable, and without pharmacological adverse effects. The latter point is especially important in older patients, who are at greater risk for adverse drug reactions. Finally, there is little published scientific evidence assessing the potential interest of hypnosis during LP, and to our best knowledge, no published study in this field includes elderly subjects [12]. The objective of this study was to examine the efficacy of hypnosis on anxiety and pain associated with scheduled LP, in a geriatric day hospital.

## Methods

### Study design and participants

A monocentric interventional randomized-controlled pilot study is conducted in university geriatric day hospital Bretonneau (Groupe Hospitalo-Universitaire Assistance Publique – Hôpitaux de Paris. Nord, Université de Paris, France), between September 20, 2019, and August 4, 2021.

The inclusion criteria were age over 70, scheduled LP for diagnostic purpose (CSF amyloid and tau biomarkers) for mild cognitive impairment to major neurocognitive disorder, speaking and understanding French, affiliation to healthcare coverage (French Social Security), and providing informed written consent to participate. The non-inclusion criteria were Mini-Mental State Examination (MMSE) score [13] below 17/30, medical contraindication to LP (e.g., space-occupying lesion with mass effect, increased intracranial pressure, anticoagulant medication), and presence of legal guardianship. The participants were randomly assigned (1:1) either to the intervention group (hypnosis, see below) or the control group (usual care) using a formatted computer-generated random list, before the beginning of the trial.

### Lumbar puncture procedure

All LP were performed using atraumatic spinal needles (Sprotte®, 22G), in a sitting position, according to the French Recommendations of the Haute Autorité de Santé for good clinical practice [14]. All the punctures were performed by a single, experienced operator, who was assisted by a trained registered nurse and a third member of the paramedical staff: either a nursing assistant in the control group or a hypnotherapist. All the members of the team ensured the person was appropriately seated in the upright position, in front of a window with a garden view. A quiet or a musical atmosphere (according to the patient’s choice) was offered to the participants in both groups, and a nurse or nurse assistant stood in front of him/her during the full procedure. The total CSF volume to be collected was 5 ml, into four tubes, for all participants.

### Hypnosis

Conversational hypnosis was performed by a nurse or a psychologist from the team, who had graduated from a training course in clinical hypnosis (2 years course of medical hypnosis at Université de Paris for the two neuropsychologists/training for Assistance Publique-Hôpitaux de Paris). The participants assigned to the intervention group followed the standardized procedure described below. In the first hour of admission in the day hospital, each participant had a 10-min consultation with the hypnotherapist during which they were informed about the principles and aims of conversational hypnosis (for additional details see Short et al. [15]). The consultation was used to answer the patient’s questions, to assess their level of anxiety, and to discover a bit about their interests (points of which may be used to formulate suggestions during the hypnosis procedure). Once in the operating room, the hypnotherapist and the patient had a more relaxed talk to help create a therapeutic link and to provide an opportunity to take note of the best sensory channels to use during the medical procedure. The induction phase started when the physician (LP operator), and the nurse went into the operating room. The patient settled down comfortably in a sitting position, focused on breathing in and out and relaxing their body. From this stage onwards, the subject only interacted with the hypnotherapist. The therapist talked with a calm voice and gave a series of direct suggestions inducing a positive response. Each response to a previous suggestion conditioned the subject to respond more strongly to the next one. This enabled the patient to reach a state of modified consciousness, and it was at this stage that the LP was performed. Meanwhile, the hypnotherapist invited the patient to enter a deeper trance state and used sensory channels to modulate the patient’s feelings (e.g., needle-related pain). Throughout, the therapist adapted to the verbal and non-verbal communication of the patient. The verbal contact was maintained using several techniques: e.g., confusion (double-negative sentences), hypnotic storytelling, diversion, never-ending sentences, and positive suggestions. The final phase of the procedure consisted of reorientation and began when the physician removed the spinal needle after collecting the cerebrospinal fluid. The hypnotherapist changed their voice tone and invited the patient to start to move freely and to come back to their natural state of awareness, before informing them that the procedure had been completed.

### Variables of interest

#### General characteristics of the population

Age, gender, educational level, body weight, cognitive performance (MMSE), and mood (15-item Geriatric Depression Scale (GDS) [16]) were specified for all participants.

Multimorbidity was assessed using the Cumulative Illness Rating Scale for Geriatrics (CIRS-G) [17]. Regarding the participants’ medication, the number of ongoing treatments, and the use of analgesics, antidepressants, anxiolytics, antipsychotics, mood stabilizers, or beta-blockers have been recorded.

#### Assessment of anxiety

Anxiety was hetero-assessed by the operator during the LP using a digital scale ranging from 0 to 10 (10 corresponds to maximum anxiety) and self-assessed by the subject with a digital scale ranging from 0 to 10 (10 corresponding to maximum anxiety).

#### Assessment of pain

Pain was hetero-assessed by the operator, during the procedure, using the Algoplus scale ranging from 0 to 5 (the value 5 corresponding to very intense pain) [18, 19]. Pain was also self-assessed with a visual-assessment scale ranging from 0 to 10 (0 meaning absence of pain and 10 highest possible pain level) [20].

#### Subjective experience of LP

The subjective experience was rated by the patient using a Likert scale. To answer the question “What was your general impression about the LP?.” The participant had the choice between five propositions: “much worse than expected,” “worse than expected,” “as expected,” “better than expected,” and “much better than expected.”

Anxiety and pain self-assessment scores as well as the patient’s subjective experience of the LP were collected during an interview conducted immediately after the procedure by another physician, blinded to the procedure. Additionally, the minimum and maximum heart rates (HR) during LP were recorded, to measure the maximum HR change over the procedure, which could reflect the sympathetic activity of the nervous system associated with anxiety or pain.

#### Variables associated with the LP procedure

Duration of the procedure (starting when the operator takes the needle until the needle is withdrawn), the number of punctures performed, and the success of the procedure (defined as the 5 ml of CSF collected) were recorded. The operator was also asked to evaluate the difficulty of the procedure (e.g., in case of scoliosis or osteoarthritis) with a numerical scale ranging from 0 to 5 (0 corresponding to “very simple” and 5 to “very difficult procedure”).

### Statistical analyses

Quantitative and qualitative variables were described using mean (± standard deviation) or percentages (numbers), respectively. Comparisons of means between the intervention group and the control group were performed using Student’s *t*-test (for Gaussian variables) or Wilcoxon test (non-parametric distribution). Percentages were compared using Yate’s chi-squared test (correction for continuity) or Fisher’s exact test, according to the sample size.

To assess the effect of hypnosis on pain and anxiety, we compared the means of the hetero- and self-report scales between the two groups. In addition, we performed a post hoc correlation test (Spearman’s correlation coefficient) between hetero-reported and self-reported anxiety and then between hetero-reported and self-reported pain. To assess the effect of beta-blocker use on the association between heart rate change and hypnosis, these variables were included in a post hoc analysis of covariance (ANCOVA).

The *α* risk was set at 0.05 for two-tailed tests. All the statistical analyses were performed using RStudio (V 1.1.463 – © 2009–2018 RStudio, Inc.).

## Results

The mean age of the participants was 77.2 (standard deviation = 5.0). The characteristics of our study population were summarized in Table 1. There was no between-group difference regarding the characteristics of the participants, except for the rate of beta-blockers prescription, which was higher in the hypnosis group (*χ*^2^ = 6.13, *p* = 0.01). The mean MMSE score was 23.2 (3.5). Self-reported anxiety and hetero-evaluated anxiety scores were significantly lower in the hypnosis group (Wilcoxon test, *W* = 413.0, *p* < 0.05, difference between medians (Hodges-Lehmann) − 1.0 95% CI [− 3.0; 0.0]; Wilcoxon test, *W* = 465.0, *p* < 0.01, difference between medians (Hodges-Lehmann) − 2.0 95% CI [− 3.0; − 1.0], respectively) than in the control group (see Table 2). The positive and significant correlation between hetero- and self-reported anxiety was moderate (Spearman’s rho = 0.55, *p* < 0.001). Hetero-evaluated pain was significantly lower in the hypnosis group (Wilcoxon test, *W* = 411.5, *p* < 0.05, difference between medians (Hodges-Lehmann) − 1.0 95% CI [− 1.0; 0.0]), whereas self-evaluated pain was not (Wilcoxon test, *W* = 386.0, *p* = 0.15). However, both showed a strong positive correlation (Spearman’s rho = 0.80, *p* < 0.001). HR variation was significantly lower under hypnosis (Student’s *t*-test, *t* = 2.4, df = 41, *p* < 0.05, Cohen’s *d* = 0.85 indicating a large effect size). After controlling for beta-blocker use (ANCOVA analysis), the difference in HR variation between the hypnosis group was still significant (*p* < 0.05).

**Table 1:** Characteristics of study population

Mean (standard deviation) or *n* (%)	Total (*n* = 50)	Hypnosis group (*n* = 25)	Control group (*n* = 25)	Test statistic	*p*-value
Age (years)	77.2 (5.0)	77.0 (5.2)	77.4 (4.8)	*W* = 324.5	0.82^a^
Female gender	27 (54.0)	15 (60.0)	12 (48.0)	*χ*^2^ = 0.32	0.57^b^
Educational level					
< Primary school certificate	7 (14.0)	4 (16.0)	3 (12.0)	–	0.34^c^
Secondary education	11 (22.0)	6 (24.0)	5 (20.0)	–	
High school	10 (20.0)	7 (28.0)	3 (12.0)	–	
University	22 (44.0)	8 (32.0)	14 (56.0)	–	
Body weight (kg)	68.7 (12.2)	70.5 (10.1)	66.9 (13.9)	*t* = − 1.07, df = 43	0.29^d^
MMSE (/30)	23.2 (3.5)	23.2 (3.2)	23.2 (3.9)	*W* = 320.0	0.89^a^
GDS (/15)	2.3 (2.0)	2.2 (2.1)	2.6 (2.0)	*W* = 235.5	0.46^a^
CIRS-G (/56)	6.2 (3.3)	6.7 (3.3)	5.6 (3.3)	*t* = –1.15, df = 47	0.25^d^
Ongoing prescription					
Number of ongoing treatments	4.3 (3.0)	4.4 (3.1)	4.1 (2.9)	*W* = 289.0	0.65^a^
Analgesic	11 (22.0)	4 (16.0)	7 (28.0)	*χ*^2^ = 1.05	0.31^b^
Antidepressants	14 (28.0)	7 (28.0)	7 (28.0)	*χ*^2^ = 0	1.00^b^
Anxiolytics	4 (8.0)	4 (16.0)	0	–	0.11^c^
Hypnotics	1 (2.0)	0	1 (4.0)	–	1.00^c^
Antipsychotics	0	–	–	–	–
Mood stabilizers	0	–	–	–	–
Beta-blockers	10 (20.0)	9 (36.0)	1 (4.0)	*χ*^2^ = 6.13	**0.01**^b^

*CIRS-G* Cumulative Illness Rating Scale-Geriatric, *df* degrees of freedom, *GDS* Geriatric Depression Scale, *MMSE* Mini-Mental State Examination

^a^Wilcoxon test

^b^*χ*^2^ test

^c^Fisher’s exact test

^d^Student’s *t*-test

**Table 2:** Assessment of anxiety and pain

	Total (*n* = 50)	Hypnosis group (*n* = 25)	Control group (*n* = 25)	Test statistic	*p*-value
Anxiety median (IQR)					
Self-assessed/10	1.5 (4.0)	1.0 (2.0)	3.0 (5.0)	*W* = 413.0	**< 0.05**^a^
Hetero-assessed/10	1.0 (2.8)	1.0 (0)	3.0 (4.0)	*W* = 465.0	**< 0.01**^a^
Pain median (IQR)					
Self-assessed/10	3.0 (4.8)	2.0 (3.0)	3.0 (4.0)	*W* = 386.0	0.15^a^
Hetero-assessed/5	2.0 (1.0)	1.0 (1.0)	2.0 (2.0)	*W* = 411.5	**< 0.05**^a^
HR change (bpm) mean (SD)	4.4 (5.7)	2.3 (4.9)	6.3 (5.9)	*t* = 2.4, df = 41	**< 0.05**^**b**^
Subjective experience of LP					
Much worse than expected	0	–	–	–	0.42^c^
Worse than expected	2 (4.0)	0	2 (8.0)	–	
As expected	14 (28.0)	7 (28.0)	7 (28.0)	–	
Better than expected	20 (40.0)	9 (36.0)	11 (44.0)	–	
Much better than expected	14 (28.0)	9 (36.0)	5 (20.0)	–	

*bpm* beats per minute, *df* degrees of freedom, *HR* heart rate, *IQR* interquartile range, *LP* lumbar puncture, *SD* standard deviation

^a^Wilcoxon test

^b^Student’s *t*-test

^c^Fisher’s exact test

Most participants (68%) reported a positive feedback about the procedure, with 40% describing the LP as “better than expected” and 28% “much better than expected.” None of them described the procedure as “much worse than expected.” The subjective experience of LP was not different between the groups.

Table 3 describes the LP procedure. Hypnosis was never interrupted in the 25 participants of the intervention group. Four LP (8%) were unsuccessful (i.e., no CSF collected). The mean duration of the LP was statistically shorter under hypnosis (*p* = 0.01).

**Table 3:** Description of lumbar puncture procedure

Mean (standard deviation) or *n* (%)	Total (*n* = 50)	Hypnosis group (*n* = 25)	Control group (*n* = 25)	Test statistic	*p*
Mean duration (min)	7.7 (5.4)	6.7 (5.8)	8.5 (5.0)	*W* = 428.0	**0.01**^a^
Number of punctures	1.5 (0.8)	1.4 (0.8)	1.6 (0.9)	*W* = 367.0	0.21^a^
Difficulty of the procedure (/5)	2.1 (1.7)	2.0 (1.7)	2.3 (1.7)	*W* = 306.5	0.50^a^
Success of the procedure, *n* (%)	46 (92.0)	24 (96.0)	22 (88.0)	–	0.61^b^

^a^Wilcoxon test

^b^Fisher’s exact test

## Discussion

This interventional pilot study, including 50 cognitively impaired elderly outpatients, referred to a day hospital for scheduled LP, demonstrated that hypnosis was significantly associated with reduced anxiety (either self-assessed or according to the operator’s judgment) and hetero-evaluated pain, due to the procedure. Besides, hypnosis was safe, well-accepted, and feasible in all the participants of the intervention group. We highlighted the statistically significant between-group differences regarding the side-effects of the LP, despite the small number of participants, as well as an overall satisfying perception of the procedure (68% perceived the procedure as better or much better than expected, in the whole sample). Therefore, hypnosis, in this context may be considered as an effective option for helping to alleviate the distress associated with uncomfortable medical procedures. It is worth noting that we observed a significant reduction of hetero-assessed pain under hypnosis, whereas self-assessed pain was not different between patients who underwent hypnosis and those in the control group. This discrepancy might be partly explained by the limited accuracy of the visual analogic scale to assess pain in cognitively impaired older adults [21]. Thus, pain could have been underestimated in the control group. Cognitive impairment has been reported as reducing the ability to self-report pain, and no specific pain-assessment tool has been validated as a gold standard in this setting [22]. However, we observed a strong positive correlation between hetero-assessed pain, by the physician, using the validated Algoplus scale, and self-reported pain in both groups. Therefore, the non-significant between-group difference in self-reported pain may rather be the consequence of a lack of power of the study due to the limited size of our population.

Apart from our study, little evidence is available regarding the potential benefits of hypnosis in cognitively impaired adults. Previously, one case report of a 61-year-old patient with AD suffering from a needle phobia highlighted the usefulness of hypnosis to help her with the LP procedure [12]. This method proved effective in the reported case, although the entire procedure lasted approximately 30 min, four times longer than the mean duration in our study. Nevertheless, the authors underlined the lack of published study in older adults, cognitively impaired patients, or patients with psychiatric conditions. Our data provide new insights on the use of hypnosis in the elderly and expand the conclusions of three previously published studies, supporting the potential interest of this tool in older adults. Hypnosis was suggested to be useful on chronic osteoarthritis pain [23]. However, the study population was younger than in our sample, with a mean age of 65 years old. Another study showed the effectiveness of hypnosis on the pain of elderly hospitalized patients [24]. The study population was older than in the latter study (mean age = 80.6), yet the participants were cognitively unimpaired. In addition, the main objective of the two latter studies was to examine whether hypnosis could reduce chronic pain and not pain induced by an invasive procedure.

In a third study, including adults aging from 18 to 92, the authors showed that hypnotizability measured with the Hypnotic Induction Profile Scale was not influenced by aging [10].

Using hypnosis in geriatrics when performing painful or anxiety-provoking procedures may offer several benefits. First of all, the absence of reported significant side effects, either in the published study or in our analysis, represents a major strength for patients at risk for iatrogenic complications. Hypnosis may represent an interesting alternative to anxiolytic or sedative treatments [25, 26]. Then, hypnosis includes a set of techniques that can be used independently of each other, which makes this tool very flexible. The hypnotic suggestions may be adapted to the subject’s personality, his/her areas of interest, or any sensory limitation [27]. The duration of the procedure can be adjusted according to the occurrence of technical issues or discomfort symptoms. Hypnosis associates different approaches (e.g., relaxation, “safe place” memories, suggestions) to induce relaxation and increase the patient’s sense of well-being. Thus, this technique can reduce anxiety by creating a state of comfortable relaxation [28, 29]. In addition, hypnosis may modify the perception of pain by modulating the state of consciousness [8]. Functional imaging techniques taught us that compared to a waking state, hypnotic trance is associated with a decrease in the functional activation of certain areas of the brain, suggesting an inhibition of peripheral perceptions or a mitigation of pain signalization [30]. Finally, practical hypnotherapy training for health professionals may involve healthcare professionals from different backgrounds: physicians, psychologists, nurses, and physical therapists. Hypnosis may be used in a large scope, for various medical procedures or medical conditions [31]. Of note, we used hypnosis in elderly subjects with mild to moderate major neurocognitive disorder. Thus, the hypnotherapists managed to recruit their attentional functions for several minutes. Our study was not powered to analyze the relationship between the importance of cognitive impairment and the effectiveness of hypnosis, but these issues may be addressed in future studies. Interestingly, the average duration of LP performed under hypnosis was shorter in the intervention group. We hypothesize that reduced pain and anxiety in the intervention group may explain the significantly lower HR variation. Likewise, the subjects of the hypnosis group might have experienced less uncontrolled movements, making the procedure easier for the operator.

LP procedure is often perceived as invasive, with a substantial risk of adverse events. Contrary to expectations, anxiety and pain were relatively low in our participants. The care taken in reception and support all along the procedure could partly explain the positive reviews of the patients. No immediate complications were described in these 50 participants. These results are consistent with the study of Paquet et al. who reported an acceptance of the procedure of 93% and a feasibility of 97% among 100 LP carried out in a French tertiary memory clinic [6]. These results confirm that CSF collection by LP may be ethically prescribed as a diagnostic procedure, in older patients with major neurocognitive disorders.

The main strength of this pilot study is the original finding suggesting that hypnosis may be effective on anxiety and pain, in older patients referred for scheduled LP. We observed a significant and clinically meaning difference supporting the use of hypnosis in this context, despite a small number of participants in both groups. The factors associated with the success of hypnosis in older adults also deserve further investigation.

Several limitations of this work must be acknowledged. The response to hypnosis is difficult to assess since this method cannot be standardized; the hypnotherapist fitted with the participant’s preferred sensory channels for inducing trance. In addition, the degree of compliance to the intervention (i.e., hypnotizability) was not assessed in our protocol. We did not assess the individual effect of each hypnotherapist on pain or anxiety. Self-assessment of pain or anxiety could be biased in cognitively impaired older individuals. However, we observed a strong association between the physician’s judgment and the patient’s assessment. Finally, our sample was small and mostly included highly educated patients with a low prevalence of multimorbidity, which could represent a selection bias. External validation would be necessary to confirm the validity of our results.

## Conclusions

This pilot study suggested that hypnosis is feasible and could be used to reduce the symptoms of discomfort due to invasive procedures in cognitively impaired older adults. Our results also underlined the overall good acceptance of LP in this population, even though this procedure had often been perceived as invasive. Hypnosis appeared as a safe and well-accepted method in this context. The use of hypnosis may be studied in broader fields: in other populations of elderly subjects or for other care or anxiety-inducing or painful procedure. Future interventional trials around this topic would be useful to better define the framework for using hypnosis in older adults.

## Data Availability

The datasets used for the analyses are available from the corresponding author on reasonable request.

## Abbreviations

AD: Alzheimer’s disease
CSF: Cerebrospinal fluid
CIRS-G: Cumulative Illness Rating Scale for Geriatrics
GDS: Geriatric Depression Scale
HR: Heart rate
LP: Lumbar puncture
MMSE: Mini-Mental State Examination
